# Cannabidiol but not cannabidiolic acid reduces behavioural sensitisation to methamphetamine in rats, at pharmacologically effective doses

**DOI:** 10.1007/s00213-022-06119-3

**Published:** 2022-04-18

**Authors:** Laísa S. Umpierrez, Priscila A. Costa, Eden A. Michelutti, Sarah J. Baracz, Melanie Sauer, Anita J. Turner, Nicholas A. Everett, Jonathon C. Arnold, Iain S. McGregor, Jennifer L. Cornish

**Affiliations:** 1 Department of Psychology, Macquarie University, North Ryde, NSW Australia; 2 Lambert Initiative for Cannabinoid Therapeutics, University of Sydney, Sydney, NSW Australia; 3 Brain and Mind Centre, The University of Sydney, Sydney, NSW Australia

**Keywords:** Cannabidiol, Cannabidiolic acid, Methamphetamine, Psychosis, Locomotor activity, Sensitisation

## Abstract

**Rationale:**

Cannabidiol (CBD) and cannabidiolic acid (CBDA) are non-psychoactive components of the cannabis plant. CBD has been well characterised to have anxiolytic and anticonvulsant activity, whereas the behavioural effects of CBDA are less clear. Preclinical and clinical data suggests that CBD has antipsychotic properties and reduces methamphetamine self-administration in rats. An animal model that is commonly used to mimic the neurochemical changes underlying psychosis and drug dependence is methamphetamine (METH) sensitisation, where repeated administration of the psychostimulant progressively increases the locomotor effects of METH.

**Objective:**

The aim of this study was to determine whether CBD or CBDA attenuate METH-induced sensitisation of locomotor hyperactivity in rats.

**Methods:**

Eighty-six male Sprague Dawley rats underwent METH sensitisation protocol where they were subjected to daily METH (1 mg/kg on days 2 and 8, 5 mg/kg on days 3–7; i.p.) injections for 7 days. After 21 days of withdrawal, rats were given a prior injection of CBD (0, 40 and 80 mg/kg; i.p.) or CBDA (0, 0.1, 10 and 1000 µg/kg; i.p.) and challenged with acute METH (1 mg/kg; i.p.). Locomotor activity was then measured for 60 min.

**Results:**

Rats displayed robust METH sensitisation as evidenced by increased locomotor activity to METH challenge in METH-pretreated versus SAL-pretreated rats. CBD (40 and 80 mg/kg) reduced METH-induced sensitisation. There was no effect of any CBDA doses on METH sensitisation or acute METH-induced hyperactivity.

**Conclusion:**

These results demonstrate that CBD, but not CBDA, reduces METH sensitisation of locomotor activity in rats at pharmacologically effective doses, thus reinforcing evidence that CBD has anti-addiction and antipsychotic properties.

## Introduction

The psychostimulant methamphetamine (METH; *Ice*) is a highly addictive illicit drug with its use producing major physical and mental health problems worldwide (Fulcher et al. 2018; Gao et al. 2018; Krizman-Matasic et al. 2019). Chronic METH use is associated with cognitive, neurological and psychiatric health problems, including drug dependence, drug-induced aggression and psychosis (Grant et al. 2012; McKetin et al. 2013; Mullen et al. 2018). METH-induced psychosis is believed to result from an excess of synaptic dopamine (DA) and psychotic symptoms are one of the most common adverse consequences among METH users. These symptoms include hallucinations and paranoid delusions (Zweben et al. 2004, McKetin et al. 2006, Glasner-Edwards and Mooney 2014), affecting up to 40% of users (Glasner-Edwards and Mooney 2014). The symptoms may re-occur spontaneously followed by re-exposure to a low dose of METH, or spontaneously after long-term cessation of METH use (Akiyama et al. 2011).

To date, there are no approved pharmacological treatments for METH dependence, withdrawal or its psychiatric sequelae (Shoptaw et al. 2009) and existing approaches (e.g. dexamphetamine substitution, antidepressants) are of minimal efficacy and have considerable adverse side effects (Morley et al. 2017). Antipsychotics aimed at dopamine systems are also associated with a number of side effects, which can be severe and may contribute to non-adherence to the treatment (Leucht et al. 2013; Davies and Bhattacharyya 2019). Due to the limitations of current treatments available for METH-induced psychoses, more research is needed to establish new pharmacotherapies, with a more favourable toxicity profile (Millan et al. 2016; Davies and Bhattacharyya 2019).

A non-intoxicating component of *Cannabis sativa*, cannabidiol (CBD) reduced seizures in childhood epilepsy patients in a series of phase III clinical trials (Thiele et al. 2018, Devinsky et al. 2018) and is now a registered therapeutic in the USA, Europe and Australia. Accumulating human research also suggests that CBD might be useful as a neuropharmacological agent in the treatment of psychiatric disorders such as depression (Resstel et al. 2009), anxiety (Masataka 2019; Linares et al. 2019) and schizophrenia (Leweke et al. 2012; McGuire et al. 2018) (Bhattacharyya et al. 2010; Fusar-Poli et al. 2010). Preclinical studies suggest that CBD may also have potential in addiction medicine to reduce the addictive effects of several abused drugs, with CBD displaying potent anti-craving effects in animal models of alcohol, cocaine and opioid addiction (Ren et al. 2009; Prud'homme et al. 2015; Gonzalez-Cuevas et al. 2018; Viudez-Martínez et al. 2018a, b). Moreover, our laboratory recently showed that treatment with CBD reduced the motivation to self-administer intravenous METH and also relapse to METH-seeking behaviour in rats (Hay et al. 2018).

Extending on this earlier finding, the current study aimed to determine whether systemic CBD treatment was effective in reducing behavioural sensitisation to repeated METH administration. Behavioural sensitisation refers to the phenomenon whereby rats given intermittent METH become progressively more hyperactive to a fixed dose of the drug, and is thought to model some aspects of METH-induced addiction and psychosis (Wearne et al. 2015; Berridge and Robinson 2011). Previous studies suggest that repeated CBD exposure attenuates dexamphetamine-induced hyperlocomotion in mice (Long et al. 2010) while intra-nucleus accumbens pretreatment of CBD reduced amphetamine-induced behavioural locomotor sensitisation in rats (Renard et al. 2016a). On the other hand, a recent study suggests that CBD may actually facilitate METH sensitisation in a conditioned place preference model (Khanegheini et al. 2021). The present study sought to clarify these disparate findings.

In the *Cannabis sativa* plant, the precursor molecule to CBD is cannabidiolic acid (CBDA). CBDA is biosynthesised enzymatically in the plant and is then decarboxylated into CBD due to exposure to heat and light. CBDA itself has emerging therapeutic properties with exhibits anti-emetic, antidepressant, anxiolytic and anticonvulsant activity, shown in various preclinical models (Hen-Shoval et al. 2018; Pertwee et al. 2018; Anderson et al. 2019a; Assareh et al. 2020). However, the knowledge of the pharmacological effects of CBDA remains rather limited. CBDA exerts its effects at much lower doses than CBD with very low microgram doses of CBDA preventing nausea-induced behaviour in rats by enhancing 5-HT1A receptor activation (Bolognini et al. 2013; Rock and Parker 2013, 2015). Further, CBDA reduced stress-induced anxiety and depression-like behaviour in rodent models (Hen-Shoval et al. 2018; Assareh et al. 2020). CBDA reduced seizures in a mouse model of Dravet syndrome at a tenfold lower doses than CBD (Anderson et al. 2019a, b). Overall, these results suggest that CBDA has common pharmacological activity to CBD, but with higher potency. We therefore aimed to compare the effects of CBD and CBDA on behavioural sensitisation to METH, to more fully characterise any potential antipsychotic and anti-addictive properties of these compounds.

## Material and methods

### Animals

Eighty-six male Sprague Dawley rats (weighing an average of 330 g upon arrival) were obtained from the Animal Resource Centre (Perth, Australia). Rats were housed in groups of four per cage (cage size: 64 × 20 × 40 cm), and food and water were available ad libitum in the home cages but not during experimental procedures. Lighting was kept on a 12-h light/dark cycle (lights on 06:00), with all experiments conducted during the light cycle. The housing room temperature was maintained at 21 °C (± 1 °C). Prior to the start of experimentation, rats were acclimatised to the facility for 7 days and were handled daily for a further 7 days. All experimental procedures were conducted in accordance with the Australian Code of Practice for the Care and Use of Animals for Scientific Purposes (8th edition, 2013) and were approved by the Macquarie University Animal Ethics Committee.

### Drug treatment

Methamphetamine hydrochloride (METH) was purchased from the Australian Government Analytical Laboratories (Pymble, NSW, Australia) and was dissolved in saline (SAL; 0.9%) for administration via intraperitoneal (i.p.) injection at doses of either 1 or 5 mg/kg. METH and SAL i.p. injections were given at a volume of 1 ml/kg. For experiment 1, CBD was purchased (THC Pharm GmbH, Germany) and suspended in a vehicle (VEH; 1:1:18 mixture of DMSO:Tween-80:SAL) and was given at doses of either 40 or 80 mg/kg at a volume of 2 ml/kg, based on our previous study (Hay et al. 2018). For experiment 2, CBDA (purity > 99%; extracted from plant material by the Lambert Initiative for Cannabinoid Therapeutics) was also prepared in DMSO:Tween-80:saline (1:1:18 ratio) and was administered (i.p.) at a dose of either 0.1, 10, or 1000 µg/kg based on dosing in previous studies (Rock et al. 2015, 2018) at a volume of 1 ml/kg. The VEH solutions (2 ml/kg for CBD and 1 ml/kg for CBDA) were administered as control treatments to compare with the effects of both CBD (experiment 1) and CBDA (experiment 2).

### Locomotor activity

**Fig. 1:**
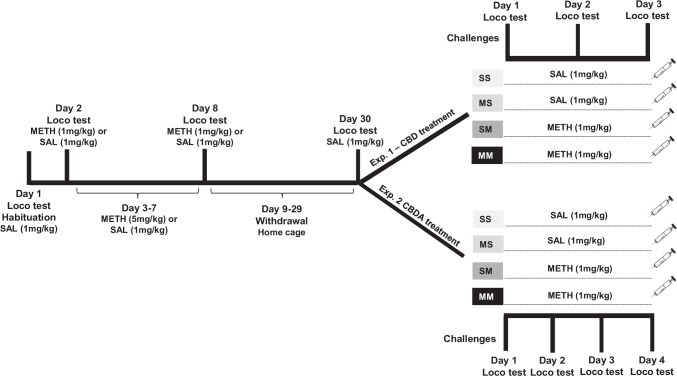
Schematic of experimental procedure and timeline for METH sensitisation protocol. Experiment 1 tested CBD treatment; experiment 2 tested CBDA treatment. Abbreviations: METH, methamphetamine; SAL, saline; VEH, vehicle; CBD, cannabidiol; CBDA, cannabidiolic acid; Loco, locomotor activity

In order to confirm the development of behavioural sensitisation and to verify the effects of CBD and CBDA, locomotor activity was recorded on days 1, 2, 8, 30, 31, and on the challenge days (Fig. 1). Locomotor activity was measured in actimeter infrared chambers (L 36 × W 24 × H 19 cm; Imetronic Pessac, France). Each chamber consisted of a removable plastic box with mesh wire top and flooring. Within each chamber were four parallel horizontal infrared sensors which recorded photocell beam breaks in three dimensions. Prior to all testing sessions, rats were placed in the test chamber for 15 min to reduce novelty-induced increases in activity before locomotor activity was recorded (60 min). Each chamber was cleaned with F10 veterinary disinfectant solution (Chemical Essentials Pty/Ltd) between trials.

### Experimental procedure

Two experiments were conducted for each treatment—experiment 1: CBD, *n* = 38 and experiment 2: CBDA, *n* = 48. Rats received either chronic SAL or METH injections during the sensitisation protocol, and then received challenge injections of either SAL or METH on test days. Within each experiment, the animals were allocated between a total of 4 groups (*n* = 9–10 rats per group for experiment 1 and *n* = 12 rats per group for experiment 2): SAL-pretreated rats + SAL challenge injection (SS), SAL-pretreated rats + METH challenge injection (SM), METH-pretreated rats + SAL challenge injection (MS) and METH-pretreated rats + METH challenge injection (MM).

Animals underwent a standard protocol for producing the expression of behavioural sensitisation to repeated METH as previously described (Wearne et al. 2015). Briefly, according with the timeline (Fig. 1), on day 1 (habituation) all animals were first acclimated to the locomotor boxes for 15 min, removed and injected with SAL (0.9%, 1 ml/kg i.p.) and replaced into the chamber for locomotor behaviour measures for a further 60 min. On days 2 and 8, rats received an i.p. injection of SAL or METH (1 mg/kg) followed by a locomotor activity test (60 min). On days 3–7, rats from the METH group received once daily i.p. injections of METH (5 mg/kg), while control rats received SAL in their home cage. Rats then underwent a 21-day withdrawal period in their home cages until the challenge tests. On day 30, all rats were injected SAL and placed to the locomotor apparatus for testing conditioned baseline response before challenge tests which began the next day and were each separated by at least 48 h (experiments 1 and 2). All treatment conditions of both CBD and CBDA were counterbalanced across the challenge test days using an adapted (CBD, 3 doses) or full (CBDA, 4 doses) Latin square design (within-subjects design), so all rats were tested on all doses of CBD or CBDA.

#### Experiment 1—CBD treatment

Rats from all 4 treatment groups received each of the three CBD treatment conditions (0 mg/kg (VEH), 40 mg/kg (CBD40) and 80 mg/kg (CBD80); i.p.) 30 min prior to the challenge dose injection of SAL or METH (1 mg/kg; i.p.) according to each treatment group assigned and were then placed into the locomotor apparatus for a total of 60 min on 3 different test days.

#### Experiment 2—CBDA treatment

All rats received all four CBDA treatment conditions (0 μg/kg (VEH), 0.1 μg/kg (CBDA0.1), 10 μg/kg (CBDA10), 1000 μg/kg (CBDA 1000); i.p.) 5 min prior to the challenge dose injection of SAL or METH (1 mg/kg), to ensure that the effects of the challenge dose coincided with the short T_max_ of CBDA (Anderson et al. 2019a). Rats were then placed into the testing chambers and locomotor activity was recorded for a total of 60 min, on 4 different test days.

### Statistical analysis

Statistical analyses were performed using SigmaPlot 12.0 to test our a priori hypotheses that either CBD or CBDA treatment would reduce METH sensitised behaviours greater than when compared to acute METH challenge, or to that of VEH-treated controls. Two-way ANOVA followed by post hoc Tukey test was used to compare the locomotor activity in the METH sensitised rats and the different CBD/CBDA doses. Two-way ANOVA repeated measures (RM) followed by post hoc Tukey was used to compare the locomotor activity between the groups and the different days. Two-way ANOVA repeated measures followed by post hoc Tukey was used to compare the effects of the different doses of CBD/CBDA over the session time within the SM and MM groups. *p* < 0.05 was considered statistically significant. Data were reported as mean ± standard error of the mean (SEM). Where interactions did not reach significance in time course data, post hoc Tukey tests were conducted to test our hypotheses.

## Results

### Sensitisation to METH

**Fig. 2:**
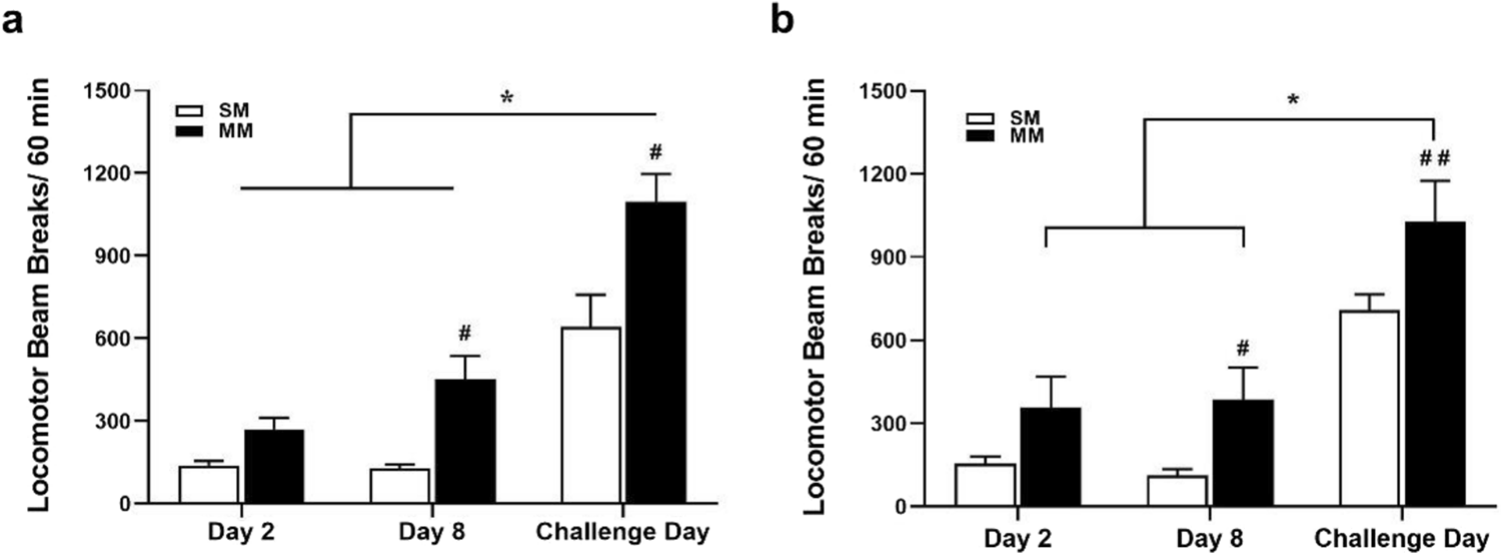
Locomotor sensitisation to repeated METH administration. Both figures represent comparisons between saline-pretreated rats (SM) and METH-pretreated rats (MM) after a METH challenge injection. **a** Experiment 1 (CBD): #*p* < 0.001 when comparing MM with SM group on challenge day and day 8; *p < 0.001 when comparing the challenge day with both day 2 and day 8 in both groups. **b** Experiment 2 (CBDA): ##*p* < 0.001 comparing MM rats with SM on challenge day, and #*p* = 0.043 on day 8; *p < 0.001 when comparing the challenge day with both day 2 and day 8 within the MM rats. (Challenge day, i.e. METH challenge 30 min after treatment with VEH)

Both experiments reproduced METH sensitisation behaviour as shown in Fig. 2a and b. Figure 2a shows that there was a main group effect [*F*(1,30) = 19.35, *p* < 0.0001], where MM group showed higher locomotion than the SM control group in the first METH challenge (*p* < 0.001) and day 8 (*p* = 0.001). A two-way RM ANOVA also revealed a day effect [*F*(2,30) = 53.41, *p* < 0.001], where both MM and SM had a significant locomotor response increase on challenge day when compared to their locomotor response on days 2 and 8 (*p* < 0.001). In Fig. 2b, a two-way RM ANOVA also revealed a significant effect of group [*F*(1,36) = 29.37, *p* < 0.001] and effect of day [*F*(2,36) = 13.08, *p* < 0.001], where the post hoc test showed that MM had higher locomotion than the SM after the first METH challenge (*p* < 0.001) and on day 8 (*p* = 0.043). In addition, a planned contrast test revealed that within MM rats on challenge day the locomotor activity was higher than on day 8 and day 2 (*p* < 0.001). Challenge day is defined as the day the rats received a challenge of METH 30 min after treatment with VEH.

### Effect of CBD on locomotor activity

**Fig. 3:**
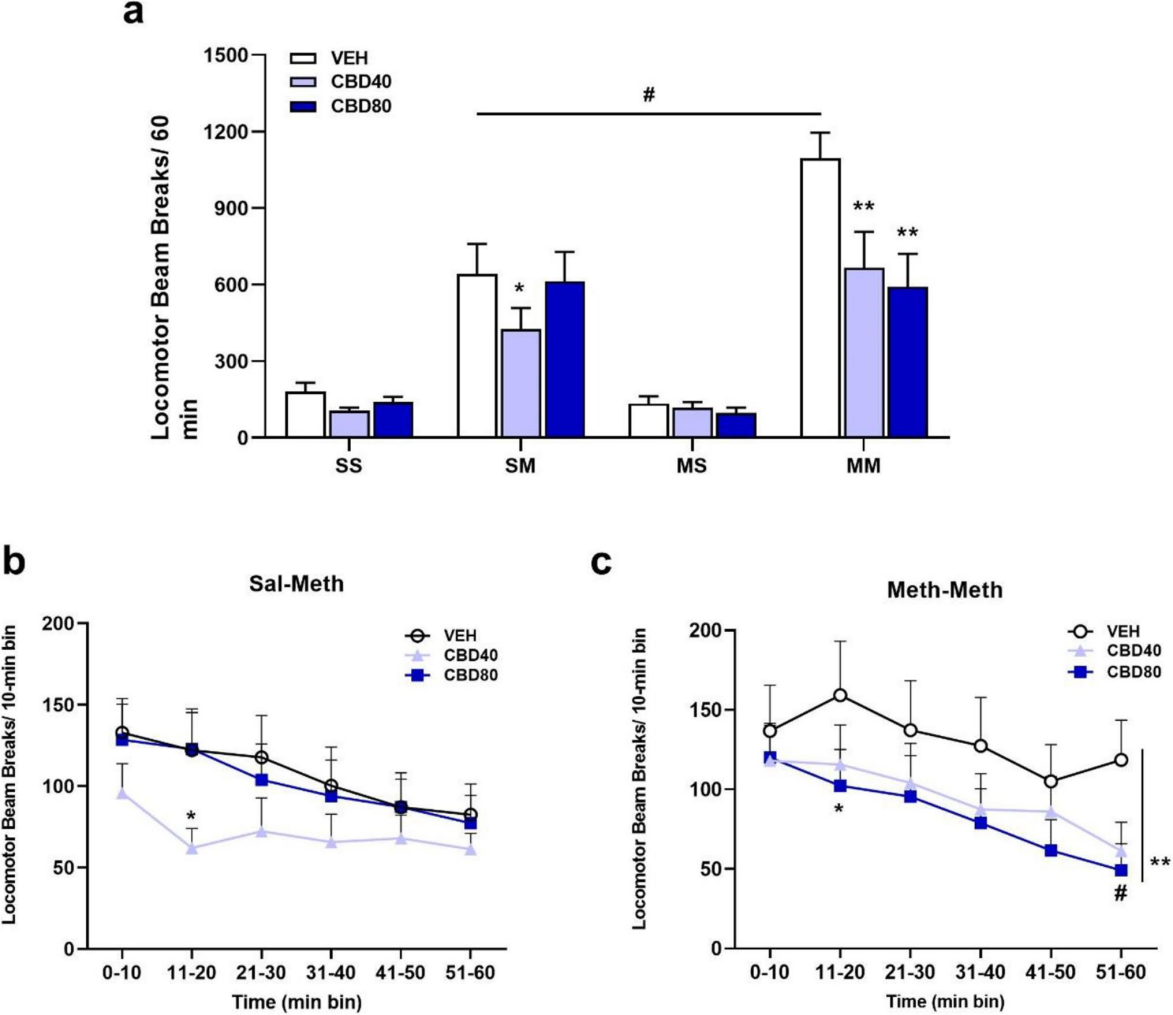
Mean (± SEM) of locomotor activity after CBD or vehicle administration on METH challenge days and number of photocell beam breaks per each 10-min bins following CBD or vehicle pretreatment. **a** **p < 0.001 when compared to rats treated with VEH in the METH-pretreated (MM) group, #*p* < 0.001 when compared within VEH treatment of METH-pretreated rats (MM) to saline control (SM) on challenge day, *p = 0.02 when compared to rats treated with VEH in the saline control (SM) group. **b** Time-course of locomotor activity within saline-pretreated rats after METH challenge injections (SM). *p < 0.05, significant difference between CBD40 and VEH/CBD80. **c** Time-course of locomotor activity within METH-pretreated rats after METH challenge injections.*p = 0.05, significant difference between CBD80 and VEH at 11–20 min time bins; **p < 0.05, significant difference of CBD40 and CBD80 from VEH at 51–60 min time bins; ^#^*p* < 0.05, significant difference between 51–60 time bins and 0–10, 11–20 and 21–30 min time bins within CBD40 and CBD80

Treatment with CBD significantly decreased the total locomotor activity in METH sensitised rats (Fig. 3a). Two-way ANOVA RM showed that there was a main effect of group [*F*(3,64) = 26.35, *p* < 0.001], CBD dose [*F*(2,64) = 11.53, *p* < 0.001] and an interaction between group and CBD dose [*F*(6,64) = 4.73, *p* < 0.001]. The post hoc test revealed a significant reduction of ambulation at both doses (CBD40 and 80) (*p* < 0.001) compared to the control group (VEH) within MM rats. However, there was no statistically significant difference between these doses (*p* = 0.710). Within the SM group, the figure displays that only the CBD40 dose was able to decrease locomotor activity when compared to VEH treatment (*p* = 0.026).

Analysing the time-course of locomotor activity, two-way ANOVA RM revealed that there was only an effect of time [*F*(5,80) = 6.26, *p* < 0.001] (Fig. 3b and c). Post hoc test showed that CBD40 significantly decreased locomotor activity in SM rats at 11–20 min time bins of the locomotor test compared to VEH and CBD80 (*p* < 0.05; Fig. 3b). In Fig. 3c, two-way ANOVA RM revealed that there was only a time effect [*F*(5,90) = 11.83, *p* < 0.001]. Post hoc tests showed that the treatment with CBD80 significantly decreased locomotor activity in MM rats at 20 min of the locomotor test compared to VEH (*p* = 0.05). The graph also shows that at 51–60 min time bins in both doses of CBD locomotor activity was decreased relative to VEH (*p* < 0.05). Within CBD40 and CBD80 there was a strong time effect, where at the 51–60 min time bins the locomotor activity was lower than at 0–10, 11–20, 21–30 min time bins (*p* < 0.05), that was not shown in VEH-treated controls.

### Effect of CBDA on locomotor activity

**Fig. 4:**
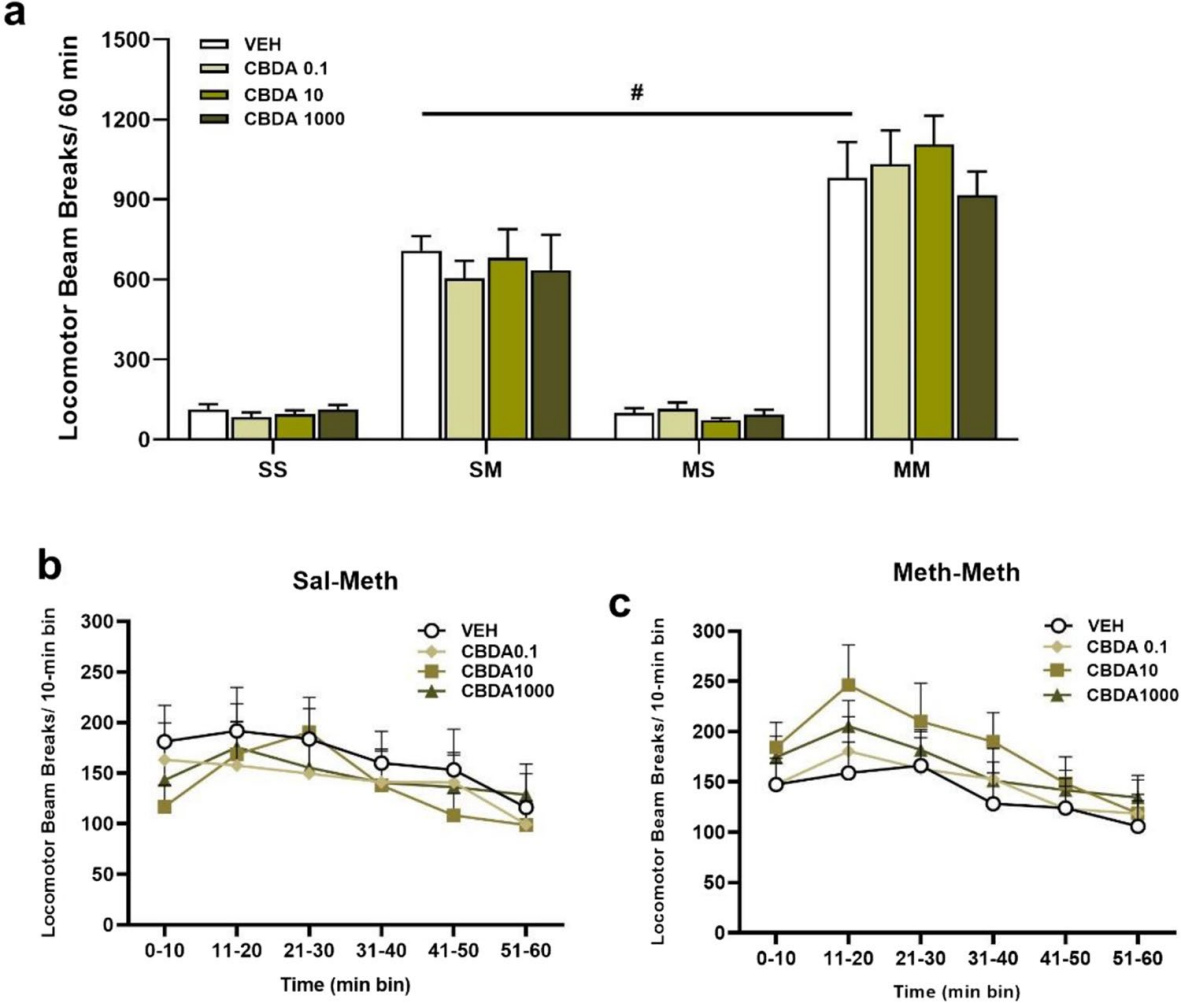
Mean (± SEM) of locomotor activity after CBDA or vehicle administration on METH challenge days and number of photocell beam breaks per each 5-min bins following pretreatment with VEH, CBDA0.1, CBDA10 and CBDA1000 for METH-pretreated group (MM) and its control (SM). **a** #*p* < 0.001 when compared the VEH from METH-pretreated rats (MM) to saline control (SM) on challenge day. **b** and **c** revealed no significant effect of CBDA on beam break activity over the 60-min test session in both SM (*p* =0.619) and MM (*p* =0.415) groups

In Fig. 4, two-way ANOVA RM showed that there was only a main effect of group [*F*(3,111) = 59.03, *p* < 0.001] and there was no effect of CBDA dose [*F*(3,111) = 0.470, *p* = 0.704] and no interaction between group and CBDA dose [*F*(9,111) = 0.602, *p* = 0.793]. Post hoc test showed only that VEH from the MM group had a higher locomotor activity when compared to its counterpart from the SM group (*p* < 0.001), indicating the sensitised locomotor effect in MM. In Fig. 4b and c, two-way ANOVA RM did not show a significant effect of CBDA on beam break activity over the 60-min test session in both SM and MM groups (SM) [*F*(3,165) = 0.601, *p* = 0.619]; (MM) [*F*(3,150) = 0.980, *p* = 0.415]; but showed a general effect of time (SM) [*F*(5,165) = 8.935, *p* < 0.001]; (MM) [*F*(5,150) = 8.613, *p* < 0.001] and an interaction between CBDA treatment and time in the SM group [*F*(15,165) = 1.804, *p* = 0.038].

## Discussion

The current study investigated the effect of CBD or CBDA treatment on the expression of behavioural sensitisation to METH, which is thought to model some aspects of METH-induced addiction and psychosis. The major findings of this study were that, in rats which underwent METH sensitisation, CBD at 40 and 80 mg/kg decreased this sensitised response. Treatment with the 40 mg/kg dose of CBD also significantly reduced the locomotor effect of METH administration in non-sensitised animals. There were no effects of treatment with CBDA on any treatment group.

The behavioural sensitisation to METH observed here is in accordance with the literature (Pierce and Kalivas 1997; Ago et al. 2012) and our previous studies (Wearne et al. 2015; Wearne et al. 2017), where repeated exposure to METH produces an enhanced locomotor response after an extended withdrawal period compared to an acute METH challenge (Robinson and Becker 1986; Vanderschuren and Kalivas 2000). This hyperlocomotion resulting from the METH sensitisation paradigm is thought to be a model of some aspects of the emergence of psychotic symptoms and METH addiction in humans that occur with repeated METH use. The ability of antipsychotics to reverse such sensitisation gives some indication of predictive validity (Jones et al. 2011; Akiyama et al. 2011). Our results also show that CBD itself did not decrease locomotion in the rats treated only with saline (SS), suggesting that its inhibitory effect on hyperlocomotion is not due to motor impairment and is consistent with numerous prior studies highlighting showing CBD does not inhibit locomotor activity in rodents (ElBatsh et al. 2012, Todd and Arnold 2016). These results agree with previous preclinical studies demonstrating that CBD reduces hyperlocomotion induced by amphetamine and ketamine (Moreira and Guimarães 2005), thus suggesting that CBD has antipsychotic-like effects without the detrimental motor side effects.

Systemic injections of either 40 or 80 mg/kg CBD prior to the METH challenge in sensitised rats significantly decreased the hyperlocomotor effects to a similar extent. This suggests limited dose-dependency of the effect of CBD in METH sensitised animals, somewhat in contrast to our prior study of METH self-administration and relapse, where only the highest dose of CBD (80 mg/kg i.p.) was effective in reducing addiction-related behaviours (Hay et al. 2018). Further, in the acute METH challenged animals in the present study (group SM), only the 40 mg/kg and not the 80 mg/kg dose of CBD effectively reduced locomotor hyperactivity. This was clearly illustrated in the time course data, where only 40 mg/kg significantly reduced the acute locomotor effect of METH at 10–20 min post challenge. The reason for this is not readily apparent. The behavioural effects of cannabinoids do sometimes follow a bell-shaped curve (for review see Blessing et al. 2015) and this may reflect the recruitment of different receptor and enzymatic targets with ascending doses of CBD.

The diverse actions of CBD on multiple receptor types (Seeman 2016; Morales et al. 2017) may also explain the differential effects of CBD in sensitised and non-sensitised animals. For example, chronic METH administration alters 5-HT_1A_ receptors, vanilloid receptor 1 (TRPV1), peroxisome proliferator-activated receptor γ (PPARγ) and dopamine D2 receptors (Ago et al. 2006; Maeda et al. 2007; Tian et al. 2010; Granado et al. 2011), providing possible avenues for CBD to have enhanced effects in sensitised animals compared to controls (Seeman 2016; Morales et al. 2017). The ability for CBD to reduce acute stimulant effects appears inconsistent, with some studies showing effective reductions after acute doses (30 and 60 mg/kg; Moreira and Guimarães 2005), and others reporting that only repeated doses of CBD are able to reduce the acute hyperactive effects of amphetamine (Long et al. 2010). On the other hand, some reported no significant effect of CBD administration on acute psychostimulant-induced behaviours (Valvassori et al. 2011). Further studies are required to determine the optimum treatment regimen and mechanism of action of CBD, following either acute or chronic treatment with METH.

The mechanisms through which CBD exerts its antipsychotic effects are still under investigation as are the brain regions involved. The dopamine-rich region of the nucleus accumbens is relevant to antipsychotic effectiveness on the positive symptoms of schizophrenia, while effects in the dorsal striatum are related to motoric side effects (Seeman 2002; Strange 2001). Several clinical and preclinical studies have revealed that CBD can strongly modulate the mesolimbic dopamine system (Bhattacharyya et al. 2010; Valvassori et al. 2011; Renard et al. 2016a). Consistent with this, direct infusion of CBD into the shell region of the nucleus accumbens reduced behavioural sensitisation to amphetamine and amphetamine-induced sensorimotor gating deficits in rats (Renard et al. 2016a). In humans, CBD normalised abnormal activity in brain structures linked to triggering psychosis in patients at high risk of psychosis (Davies and Bhattacharyya 2019; Allen et al. 2016; Bhattacharyya et al. 2018). Notably, Seeman (2016) showed CBD is a partial agonist at dopamine D2 receptors, behaving in a similar manner to the antipsychotic drug aripiprazole. Thus, this pharmacological property of CBD might subserve its ability to reduce METH-induced behavioural sensitisation (Seeman 2016).

In contrast to CBD, CBDA treatment did not reduce the hyperlocomotion caused by METH sensitisation in the present study. Our prediction that CBDA might inhibit the development of sensitisation to METH was partly based on the strong 5-HT_1A_ receptor agonist effects of this cannabinoid (Bolognini et al. 2013; Rock and Parker 2013, 2015), given that 5-HT_1A_ receptor agonists are known to inhibit the expression of METH sensitisation (Ago et al. 2006). Surprisingly, however, this hypothesis was not supported by the current findings where CBDA failed to modulate METH-induced behavioural sensitisation. This lack of effect of CBDA could be due to other differences in the pharmacological targets of CBD and CBDA. Moreover, the dose range employed here was much lower for CBDA than CBD, although the doses chosen were based on CBDA’s potent anti-emetic and anxiolytic effects in rats and mice (Pertwee et al. 2018; Rock and Parker 2013, 2015, Rock et al. 2015; Assareh et al. 2020). Future studies might examine higher doses, given that CBDA was anticonvulsant at ≥10 mg/kg in a mouse model of childhood epilepsy (equivalent to a 5 mg/kg dose in rats) (Anderson et al. 2019a). In our experiments, CBDA did not affect baseline activity levels at any dose tested. This is consistent with a previously published report showing that CBDA does not suppress spontaneous locomotor activity at doses ≤ 1 mg/kg (Rock et al. 2014).

Comparisons of activity across the 60-min time course also revealed differences in activity of CBD and CBDA treatment on METH-pretreated rats. While CBD 40 and 80 mg/kg were able to significantly decrease locomotor activity, especially at the 50–60 min part of the test when compared to VEH, all doses of CBDA failed to significantly change behaviour throughout the entire session. It is known that the levels of CBD in the rat brain have a T_max_ of 120 min following intraperitoneal administration according to Deiana et al. (2012). In line with this, our results show that 90 min after the injection there was still a strong effect of both CBD doses in suppressing locomotor activity in the latter part of the 1-h session when compared to the 0–30-min bin.

This work further underlines the promising therapeutic potential of CBD on METH-induced addiction and psychosis. One limitation of the current data is the use of a within-subject design for challenge testing where rats were exposed to all examined doses of CBD. While the effects of CBD were controlled for by counterbalancing doses and allowing at least 48 h between tests, the repeated exposure to METH in the acute METH group may have initiated some sensitisation. However, when examining the data, it is clear that the acute METH effects were significantly lower than the sensitised group, providing confidence in our data. Further studies should explore the effect of chronic administration of CBD on METH sensitisation, given that a recent study showed an effect of chronic CBD administration to prevent the development of cocaine sensitisation in a conditioned place preference (CPP) paradigm (Chesworth and Karl, 2020). However, others report an effect of chronic CBD administration to reduce CPP, but not behavioural sensitisation to cocaine (Luján et al. 2018). Furthermore, considering that sex differences exist between males and females for the expression of cocaine sensitisation (Hu and Becker 2003), it is relevant for future studies to test the effect of CBD or CBDA treatment on METH sensitised responses in female rats. It is also important to acknowledge that the sensitisation protocol presents some advantages and disadvantages for modelling addiction and psychosis that should be considered. On the one hand, the protocol uses simple sub-chronic drug delivery to produce enduring effects on reward circuity, however the face validity is impacted by experimenter delivered drug of limited amount, and does not model the social constructs surrounding human drug use (Kuhn et al 2019). Despite these considerations, the model provides important basic knowledge to further medications discovery.

In summary, the present study showed a sensitised locomotor response to METH in male rats pretreated and that CBD, but not CBDA treatment, was able to attenuate hyperlocomotion characteristic of METH sensitisation, at pharmacologically effective doses. Future studies should explore the neuropharmacological mechanisms and associated brain circuitry involved in these effects of CBD. The data further reinforce the view that CBD might serve as a novel pharmacotherapy for METH-induced addiction and psychosis.
